# Supplementary material for: In Vitro Hepatitis C Virus Infection and Hepatic Choline Metabolism
Source: Viruses. 2020 Jan 16;12(1):108. doi: 10.3390/v12010108 (PMC7019665; doi:10.3390/v12010108)

viruses

Supplementary Table S1. qPCR primer sequences.

Gene	*Forward Primer*	*Reverse Primer*
SLC44A1	TTTCCTGCTATGCCAAGTTTGC	CCAGAATGGTTAAGATCCACACA
SLC44A2	AAAGGGAGGGAGAGTTTTGC	CCCTTGGGTGGGTTTAGTTT
SLC44A3	GTCCAAAAGCAGACTCACTGT	GCAAATAGGGAGTAGCACTCAGG
SLC44A4	GGGATCAGCGGTCTTATTGA	GCGCAGAAGCAAGATAAAC
SLC44A5	ACCCCAGAAGAGCAGCCTAT	TTTAGCAACACGGAGGGACT
SLC22A1	TAATGGACCACATCGCTCAA	ACCCCTGATAGAGCACAGA
SLC22A2	AAGAATGGGGAATCACAATGG	AGATGTGGACGCCAAGATTC
SLC5A7	TTGGTGGCCGAGATATTGGTT	GCCATTGATATACCCTCCTCCG
PCYT1A	CTCTGATGCAAGCGAAGAACC	ATCACCGTGAAGCCTTTGAAG
CHKA	CTTGGTGATGAGCCTCGGAA	AAGTGACCTCTCTGCGAGAA
CHKB	AGTCTCGGTTCCAGTTCTAC	CTTCTGCTCGTTGTTCCTCC
β ACTIN	CCAACCGCGAGAAGATGACC	GGAGTCCATCACGATGCCAG
HSP90	CCAGTTTGGTGTCGGTTTCTAT	CTGGGTATCGTTGTTGTGTTTTG
JFH1 Hepatitis C Virus	GTCTGCGGAACCGGTGAGTA	GCCCAAATGGCCGGGATA

Supplementary Figure S1. FBS-cultured Huh7.5 choline transporter transcript expression

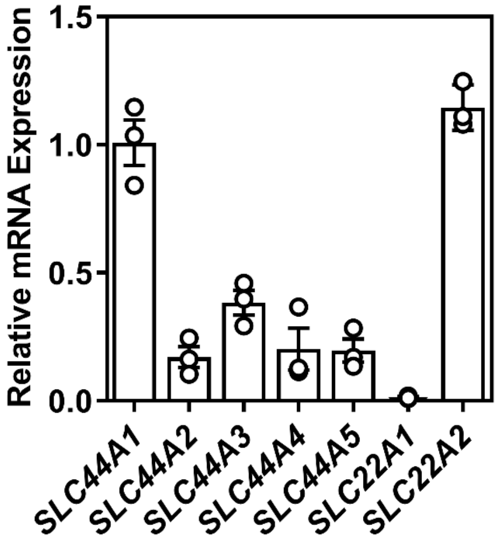

Supplementary Figure S1. FBS-cultured Huh7.5 choline transporter transcript expression. RNA was isolated from FBS-cultured Huh7.5 cells to measure choline transporter (*SLC44A1-5*; choline transporter-like 1-5, and SLC22A1 and 2; organic cation transporter 1 and 2) transcript expression. All groups are shown relative to the expression of *SLC44A1* and normalized to the average of *β ACTIN* and *HSP90*.

Supplementary Figure S2. HS-cultured Huh7.5 choline uptake kinetics

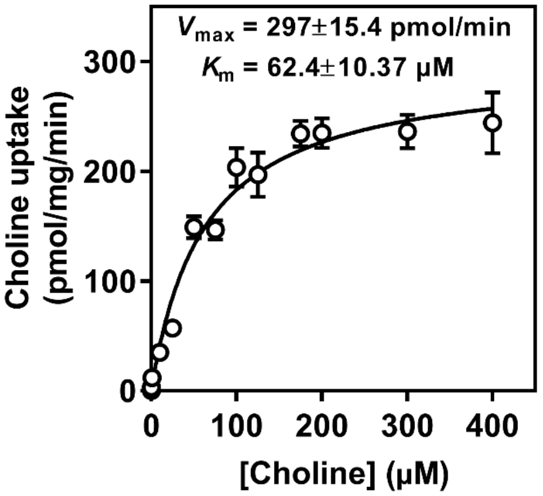

Supplementary Figure S2. HS-cultured Huh7.5 choline uptake kinetics. Choline uptake saturation kinetics from HS-cultured Huh7.5 cells, fit to a Michaelis-Menten curve. Data are mean±SEM and are representative of 3 independent experiments.

Supplementary Figure S3. Inhibition of choline transport in FBS-cultured infected cells

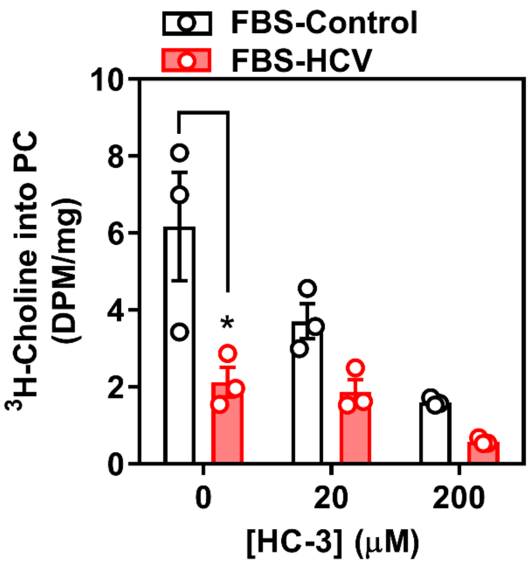

Supplementary Figure S3. Inhibition of choline transport in FBS-cultured infected cells. FBS-cultured Huh7.5 cells were infected at an MOI of 1 for 24 h in the presence or absence of 20 or 200 μM hemicholinium-3 (HC-3) to inhibit choline uptake. [^{3}H]-choline incorporation into PC was measured. Data are mean±SEM and are representative of 3 independent experiments. Statistical significance was determined by two-way ANOVA with a Tukey *post-hoc* analysis, such that * represents $p<0.05$ compared to uninfected vehicle control within inhibitor condition.

Supplementary Figure S4. Graphical abstract

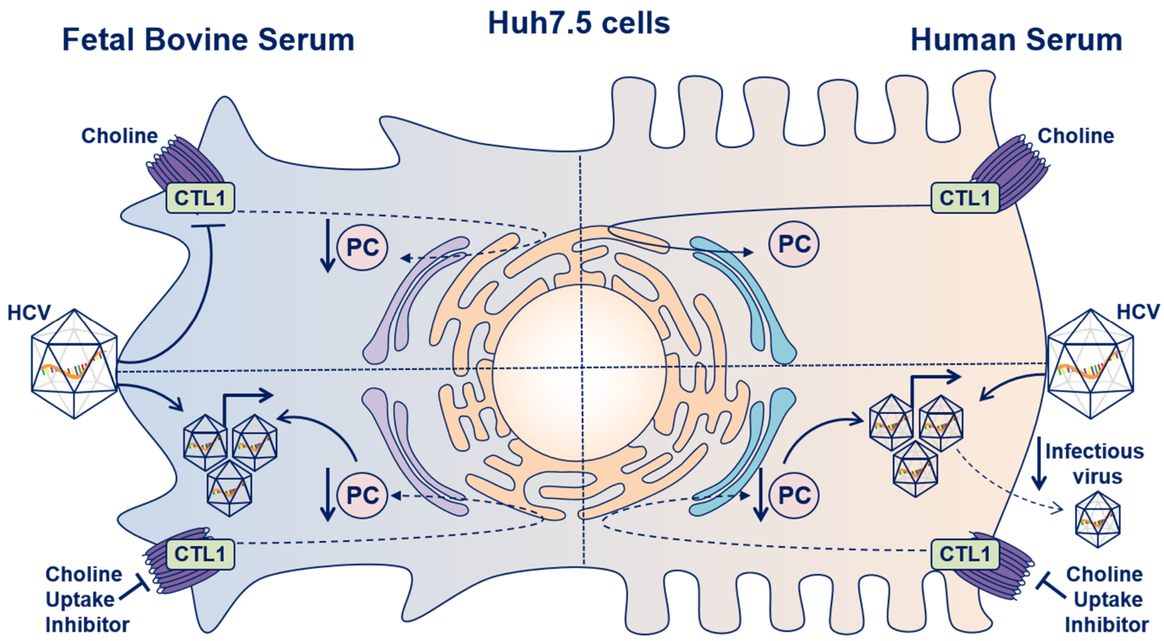

Supplement: Supplementary file 1 [file viruses-12-00108-s001.pdf]
